# Genotypes and serotype distribution of macrolide resistant invasive and non- invasive *Streptococcus pneumoniae *isolates from Lebanon

**DOI:** 10.1186/1476-0711-11-2

**Published:** 2012-01-16

**Authors:** Nedal Taha, George F Araj, Rima H Wakim, Souha S Kanj, Zeina A Kanafani, Ahmad Sabra, Marie-Therese Khairallah, Farah J Nassar, Marwa Shehab, Maysa Baroud, Ghassan Dbaibo, Ghassan M Matar

**Affiliations:** 1 Department of Experimental Pathology, Immunology & Microbiology, Faculty of Medicine, American University of Beirut, Riad El-Solh, Beirut, P.O. Box 11-0236, Lebanon; 2 Department of Pediatrics & Adolescent Medicine, Faculty of Medicine, American University of Beirut, Riad El-Solh, Beirut, P.O. Box 11-0236, Lebanon; 3 Department of Pathology & Laboratory Medicine, Faculty of Medicine, American University of Beirut, Riad El-Solh, Beirut, P.O. Box 11-0236, Lebanon; 4 Department of Internal Medicine, Faculty of Medicine, American University of Beirut, Riad El-Solh, Beirut, P.O. Box 11-0236, Lebanon; 5 Infectious Diseases Research Core Facility, Faculty of Medicine, American University of Beirut, Riad El-Solh, Beirut, P.O. Box 11-0236, Lebanon

**Keywords:** Antimicrobials, Macrolides, Resistance, Genes, Serotyping

## Abstract

**Background:**

This study determined macrolide resistance genotypes in clinical isolates of *Streptococcus pneumoniae *from multiple medical centers in Lebanon and assessed the serotype distribution in relation to these mechanism(s) of resistance and the source of isolate recovery.

**Methods:**

Forty four macrolide resistant and 21 macrolide susceptible *S. pneumoniae *clinical isolates were tested for antimicrobial susceptibility according to CLSI guidelines (2008) and underwent molecular characterization. Serotyping of these isolates was performed by Multiplex PCR-based serotype deduction using CDC protocols. PCR amplification of macrolide resistant *erm *(encoding methylase) and *mef *(encoding macrolide efflux pump protein) genes was carried out.

**Results:**

Among 44 isolates resistant to erythromycin, 35 were resistant to penicillin and 18 to ceftriaxone. Examination of 44 macrolide resistant isolates by PCR showed that 16 isolates harbored the *erm*(B) gene, 8 isolates harbored the *mef *gene, and 14 isolates harbored both the *erm*(B) and *mef *genes. There was no amplification by PCR of the *erm*(B) or *mef *genes in 6 isolates. Seven different capsular serotypes 2, 9V/9A,12F, 14,19A, 19F, and 23, were detected by multiplex PCR serotype deduction in 35 of 44 macrolide resistant isolates, with 19F being the most prevalent serotype. With the exception of serotype 2, all serotypes were invasive. Isolates belonging to the invasive serotypes 14 and 19F harbored both *erm*(B) and *mef *genes. Nine of the 44 macrolide resistant isolates were non-serotypable by our protocols.

**Conclusion:**

Macrolide resistance in *S. pneumoniae *in Lebanon is mainly through target site modification but is also mediated through efflux pumps, with serotype 19F having dual resistance and being the most prevalent and invasive.

## Background

*Streptococcus pneumoniae *continues to be a major cause of morbidity and mortality in humans. It is one of the most significant bacterial pathogens causing community acquired infections, most notably pneumonia, otitis media, bacteremia, and meningitis [1,2]. Treatment of pneumococcal infections is becoming difficult due to the high prevalence of penicillin-resistant strains and to the rapid development of resistance to other antimicrobials including macrolides. These drugs are extensively used for the treatment of respiratory infections due to their broad-spectrum of activity and safety profile. Although macrolide resistance varies geographically, it is widely spread all over the globe [3-6].

Macrolide resistance in *S. pneumoniae *is primarily due to two mechanisms; target site modification and efflux pump expulsion. Target site modification is encoded by the *erm*(B) gene which leads to reduction in the binding affinity of all macrolides to the 23S rRNA (domain V). This mechanism relies on methylation of specific adenine residues (A2058) in 23S rRNA by the methylase-product of the *erm *gene leading to cross resistance to macrolides, lincosamides, and streptogramins. Therefore, isolates harboring the *erm *gene have the MLSb (resistance to macrolides, lincosamides, and streptogramins) phenotype [7,8]. While isolates harboring the *erm *gene are resistant to all macrolides, isolates expressing an efflux pump encoded by the *mef *gene are resistant to only 14 and 15-membered macrolides. Isolates harboring the *mef *gene have the M (resistance to macrolides) phenotype [9,10].

National and regional data about serotype distribution has been very useful in vaccine introduction. The use of the seven-valent pneumococcal conjugate vaccine (PCV7) resulted in a dramatic decline in invasive pneumococcal disease in children. More recently however, a 10-valent (PCV10) and 13-valent vaccine (PCV13) were introduced after increasing reports of non-PCV7 serotypes [11,12]. Therefore, ongoing serotype surveillance is essential for evaluation of the impact and the suitability of available vaccines and their coverage in different geographic locations. Vaccination with protein-conjugated vaccines is also important in preventing the spread of vaccine-type, antibiotic-resistant strains due to its ability to significantly decrease nasopharyngeal colonization in vaccinated children [2].

Since macrolide resistance is being encountered at different medical centers in Lebanon, this study was warranted to: 1) determine the macrolide resistance genotypes among *S. pneumoniae *clinical isolates collected from medical centers in Lebanon, and to 2) assess the serotype distribution among these isolates in relation to the mechanism(s) of resistance and invasiveness of the isolates.

## Methods

### Source and Identification of *S. pneumoniae *Isolates

Sixty five *S. pneumoniae *isolates were collected from various clinical specimens including blood, sputum, bronchial wash, cerebrospinal fluid, deep tracheal aspirate, pleural fluid and other sites. Isolates were collected prospectively from various medical centers in Lebanon during the specified period of 2008 and 2010. Preliminary susceptibility testing and phenotypic identification was performed at the enrolled medical center laboratories. Subsequently, the samples were forwarded to the clinical microbiology lab at AUBMC for further characterization. Those samples determined as erythromycin resistant, and those also collected and determined at the clinical microbiology laboratory as erythromycin sensitive (2008 CLSI guidelines) were coded and sent to the centralized lab at the Department of Experimental Pathology, Microbiology and Immunology at AUB for the MSc student candidate to work on blindly [13].

### Antimicrobial Susceptibility Testing

All isolates were tested against erythromycin, tetracycline, trimethoprim-sulfamethoxazole and chloramphenicol by disc diffusion method according to the 2008 Clinical Laboratory Standards Institute (CLSI) guidelines. In addition, isolates resistant to erythromycin were further tested for susceptibility to penicillin and ceftriaxone by E-tests and results were interpreted according to the 2008 CLSI guidelines. For non-meningitis isolates an MIC ≥ 8 μg/ml for penicillin and an MIC ≥ 4 μg/ml for ceftriaxone was considered resistant. For meningitis isolates an MIC ≥ 0.12 μg/ml for penicillin and an MIC ≥ 2 μg/ml for ceftriaxone was considered resistant. Susceptibility testing was monitored by using a quality control strain (*S*. *pneumoniae *ATCC 49619) in the test runs and was verified based on the CLSI-QC breakpoint limits for this strain [14].

### Detection of Erythromycin Resistance Genes

Total DNA was extracted from all isolates using the Illustra Bacteria Genomic Prep Mini Spin Kit (GE, Healthcare, UK). Polymerase chain reactions were used to amplify two macrolide resistance encoding genes: *erm*(B) and *mef *using specific primers [15]. PCR mix consisted of 1 μM of each primer, 1× Taq buffer, 1.25 U Taq DNA polymerase, 2.0 mM of MgCl_2, _0.05 mM of deoxynucleoside triphosphate (dNTP), and 29.25 μl nanopure water. A thermal cycler (Bio-Rad, C-1000, USA) was used for amplification with PCR conditions as described by Sutcliffe et al [16]. A reference CDC *S. pneumoniae *strain harboring both the *erm*(B) and *mef *genes was used as a positive control for the PCR reactions.

### Capsular Multiplex PCR Serotype Deduction

Capsular serotyping was done by a multiplex PCR assay [17]. A total of 41 primer pairs were used and grouped into seven multiplex reactions based on serotype distributions among invasive pneumococci recovered by the United States Centers for Disease Control & Prevention [17]. Forty three CDC *S. pneumoniae *isolates with known serotypes were used as positive controls. Each reaction included a set of primers targeting different serotype specific sequences in addition to an internal positive control for a conserved region in the pneumococcal *cps *operon. Multiplex PCR serotype deduction was performed using the method described by Pai *et al*. [17]. Serotypes obtained at our lab were confirmed by both classical capsular methods and multiplex PCR at the United States Naval Medical Research Unit #3 in Cairo, Egypt.

## Results

### Antimicrobial Susceptibility

Forty four (67.7%) *S. pneumoniae *isolates were resistant to and 21 (32.3%) were susceptible to erythromycin. Susceptibility profiles of *S. pneumoniae *isolates to chloramphenicol (C), trimethoprim-sulfamethoxazole (SXT), and tetracycline (TE) are shown in Table 1. In addition, the percent susceptibility to ceftriaxone and penicillin among these isolates was 59.09% (n = 26) and 20.45% (n = 9) respectively. Fourteen of 44 isolates (31.81%) exhibited resistance to penicillin, whereas 21 of 44 isolates (47.72%) exhibited intermediate resistance to penicillin. As for ceftriaxone, 5 of 44 (11.36%) isolates were found to be resistant and 13 of 44 (29.55%) were of intermediate resistance (Table 1).

**Table 1:** Antimicrobial susceptibility profiles for 44 macrolide resistant *S. pneumoniae *isolates by disk diffusion or E-test

	Number & Percentage of Isolates		
**Antimicrobial test**	**Susceptible**	**Intermediate**	**Resistant**

**Disk Diffusion**			

Chloramphenicol (C)	n = 43 (97.72%)	n = 0 (0%)	n = 1 (2.27%)

Sulfamethoxazole-Trimethoprim (SXT)	n = 12 (27.27%)	n = 1 (2.27%)	n = 31 (70.45%)

Tetracycline (TE)	n = 10 (22.72%)	n = 1 (2.27%)	n = 33 (75%)

**E-test**			

Penicillin (PG)	n = 9 (20.45%)	n = 21 (47.73%)	n = 14 (31.82%)

Ceftriaxone (TX)	n = 26 (59.09%)	n = 13 (29.55%)	n = 5 (11.36%)

### PCR Amplification of Resistance Genes

**Figure1:**
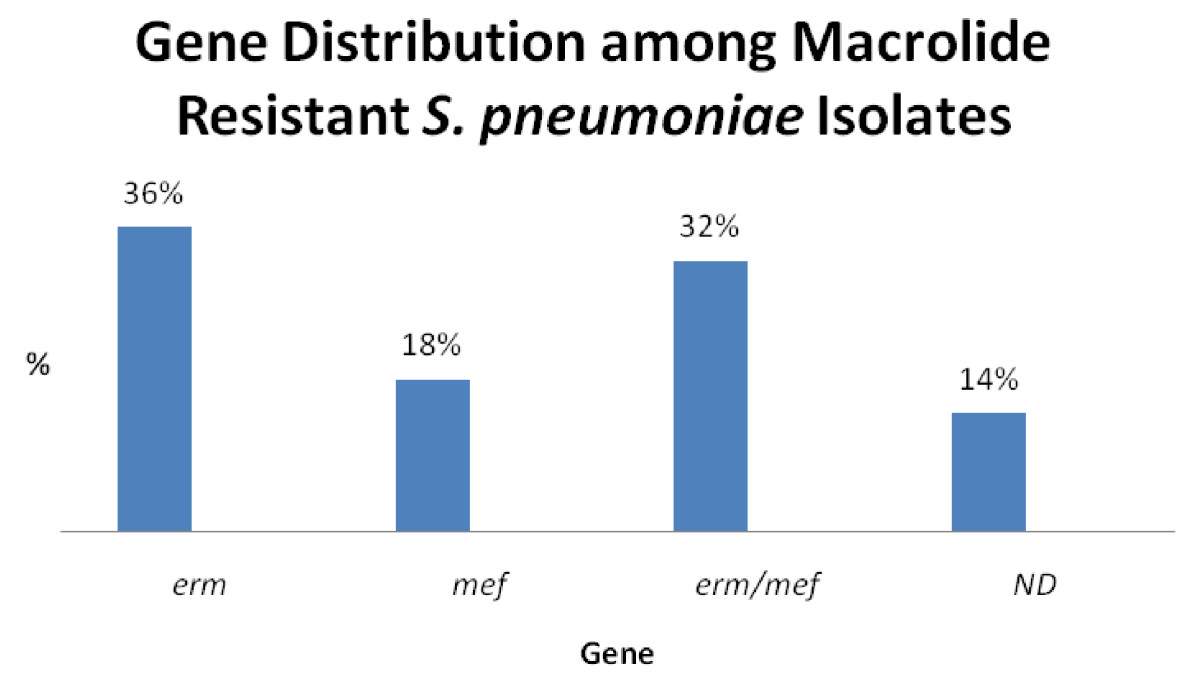
**Percentage of gene distribution among macrolide resistant *S. pneumoniae *isolates (n = 44)**. *erm: *erythromycin resistance methylase, *mef*: macrolide efflux pump, ND: not determined.

PCR amplification of the resistance encoding genes tested showed that the *erm*(B) and *mef *genes were present in 16/44 (36%) and 8/44 (18%) of *S. pneumoniae *isolates resistant to erythromycin, respectively. Fourteen of 44 (32%) isolates harbored both genes. Neither *erm*(B) nor *mef *were detected in 6/44 (14%) of the macrolide resistant isolates (Figure 1). Also, neither gene was detected in the control *S. pneumoniae *macrolide susceptible isolates.

### Multiplex PCR-Based Serotype Deduction

The resistant isolates belonged to seven different capsular serotypes: 19F (31.8%), 23 (13.6%), 2 (11.3%), 14 (9%), 19A (6.8%), 12F (4.5%), and 9V/9A (2.7%) in 35 of 44 (79.5%) isolates. Nine of the 44 isolates (20.4%) were not serotypable by our protocols. Among the 21 erythromycin susceptible isolates, 9 different serotypes were detected with 4 isolates belonging to serotype 5, 3 isolates to serotype 9V/9A, 2 isolates to serotype 6A/B/C, 3 isolates to serotype 2, and one isolate each to serotypes 15B/15C, 4, 21, 38, and 35 (Table 2).

**Table 2:** Serotype distribution among macrolide susceptible (n = 21) and macrolide resistant (n = 44) *S. pneumoniae *isolates

Serotype	Macrolide Resistant Isolates	Macrolide Susceptible Isolates
**19F**	31.8%	-

**23**	13.6%	-

**2**	11.3%	14.28%

**14**	9%	-

**19A**	4.5%	-

**12F**	2.7%	-

**9V/9A**	-	14.28%

**5**	-	19.04%

**6A/B/C**	-	9.5%

**15B/C**	-	4.76%

**4**	-	4.76%

**21**	-	4.76%

**38**	-	4.76%

**35**	-	4.76%

**No serotype detected**	20.45%	19.04%

## Discussion

This study demonstrated that two mechanisms are involved in macrolide resistance among *S. pneumoniae *isolates from Lebanon, namely, efflux pump mediated resistance and ribosomal modification due to adenine-dimethylase, with dominance of the latter. This observation is not concordant with that seen in some European countries like France, Spain, and Poland where macrolide resistance due to efflux pumps is almost exclusive [18]. This mechanism is also mostly prevalent in the USA and some other European countries like Greece and Germany [18,19]. Moreover, a high rate of dual resistance was detected in our isolates where 14 of 44 (32%) of the isolates carried both genes.

Some of our clinical isolates were found to be both *erm*(B) and *mef *negative, suggesting the possibility of one of the newly described resistance mechanisms, such as mutations in the 23S rRNA or alteration of the ribosomal proteins L4 and L22 [20,21] requiring further investigation. The high (79.5%) prevalence rate of penicillin resistance among our erythromycin resistant isolates denotes that in our community, the evolution of erythromycin resistance is driven, possibly by the spread of penicillin resistant clones since isolates with the same serotypes manifested resistance to both penicillin and macrolides. Moreover, the high rate of macrolide resistance was accompanied by a high rate of tetracycline resistance (77%) indicating a possible association to the conjugative transposon Tn1545 that confers resistance to tetracycline via the *tet*(M) gene in addition to resistance to macrolides [22].

There is increasing evidence that macrolide resistance may result in clinical failure. Studies worldwide have shown that the frequency of this resistance might be related to the level of macrolide consumption [23,24]. The same rationale may apply to Lebanon where overuse of macrolides may contribute to the observed increase in resistance to these antimicrobial agents in *S. pneumoniae*.

As already mentioned, the majority of macrolide resistant isolates in our community belonged to seven different serotypes: 19F, 23, 2, 14, 19A, 12F, and 9V/9A. These results are concordant with the most common serotypes found in Asia, Turkey, and Saudi Arabia [25-27]. The serotypes of isolates recovered from invasive pneumococcal infection cases were 19F, 14, 23, 19A, 9V/9A, and 12F with serotype 19F being the most common. Nine of 14 (64.28%) isolates carrying both the resistance genes were serotype 19F (Table 3). This might suggest that isolates belonging to serotype 19F are highly resistant to macrolides. In addition, the serotypes 12F and 14 were associated with both dual resistance and invasiveness. Serotype 2, one of the most prevalent serotypes, seems to be non-invasive since it was found among the upper and lower respiratory tract specimens, but not among specimens from sterile sites.

**Table 3:** Distribution of macrolide resistant genotypes versus serotypes among *S. pneumoniae *isolates

No. (%) of Isolates with Genotype				
	
Serotype	*erm*	*mef*	*erm *+ *mef*	ND
19F	0 (0%)	3 (21.43%)	9 (64.28%)	2 (14.29%)

23	3 (50%)	1 (16.67%)	0 (0%)	2 (33.33%)

2	3 (60%)	1 (20%)	1 (20%)	0 (0%)

14F	3 (75%)	0 (0%)	1 (25%)	0 (0%)

19A	3 (100%)	0 (0%)	0 (0%)	0 (0%)

12F	0 (0%)	0 (0%)	2 (100%)	0 (0%)

9V/9A	0 (0%)	1 (0%)	0 (0%)	0 (0%)

NS	5 (55.56%)	2 (22.22%)	0 (0%)	2 (0%)

NS: non-serotypable, ND: no amplification of *erm *and/or *mef*

It is noteworthy that the pool of serotypes found among macrolide resistant isolates was completely different from the pool of serotypes found for susceptible isolates with the exception of serotype 9V/9A which was common among macrolide resistant and macrolide susceptible isolates. Interestingly, serotype 19F, which was the major serotype found among resistant and invasive isolates, was not found among the susceptible ones. This denotes that a few, specific serotypes are responsible for macrolide resistance among our *S. pneumoniae *isolates.

Alarmingly, some of the most prevalent macrolide resistant serotypes recovered, including serotypes 2 and 12, are not covered by PCV7, PCV10 or PCV13 vaccines [12] and thus, constitute a risk for dissemination in the community. Notably the remaining macrolide resistant serotypes that were recovered are covered by PCV-7, PCV-10 and/or PCV-13, including the highly prevalent serotypes 19F, 23 and 14 [12]. In Lebanon, pneumococcal conjugate vaccines are available in the private sector but are not included in the Expanded Program of Immunization. The estimated PCV vaccine coverage is 10-15% for children under 5 years of age. Most of our isolates with PCV covered serotypes were obtained from unvaccinated subjects.

In conclusion, though the macrolide resistance observed in our *S. pneumoniae *isolates is mostly due to target site modification by the methylase encoded by the *erm*(B) gene, many isolates also demonstrated efflux pump mediated resistance to macrolides. Moreover, a number of isolates were invasive and macrolide resistant, with only a few belonging to serotypes covered by currently available vaccines. Understanding the mechanisms of resistance to macrolides may be helpful in choosing the correct treatment regimen in certain situations but is definitely important in the development of new antimicrobial agents. Moreover, continued surveillance for changes in serotype distribution is necessary, especially after the introduction of new vaccines.

## Competing interests

The authors declare that they have no competing interests.

## Authors' contributions

NT designed experimental settings, performed experiments and participated in drafting the manuscript, GFA provided clinical isolates and phenotypic testing, RHW, SSK, ZAK delivered clinical support, AS, MTK, MS, FJN, MB carried out technical help, GD and GMM conceived and designed the study, monitored the progress and supervised and drafted the manuscript. All authors have read and approved the final manuscript.
